# Molecular Identification of Spotted Fever Group Rickettsiae in Ticks in the Republic of Korea

**DOI:** 10.3390/pathogens13070575

**Published:** 2024-07-10

**Authors:** Ji-Ye Seo, Jin-Seo Park, Hee-Il Lee, Jung-Won Ju

**Affiliations:** Division of Vectors and Parasitic Diseases, Korea Disease Control and Prevention Agency, 187 Osongsaengmyeong 2-ro, Osong-eup, Heungdeok-gu, Cheongju 28159, Chungbuk, Republic of Korea

**Keywords:** tick, tick-borne rickettsiosis, spotted fever group rickettsiae, Republic of Korea

## Abstract

The Rickettsia species transmitted by ticks are mostly classified within the spotted fever group rickettsiae (SFGR), which causes tick-borne rickettsiosis. Although efforts have been made to investigate their prevalence in the Republic of Korea (ROK), research has been limited to certain areas. Furthermore, the pooling method for ticks does not fully reflect the exact infection rate. Therefore, we aimed to perform molecular identification of SFGR in ticks to elucidate the current prevalence of tick-borne rickettsiosis in the ROK. The SFGR of ticks was identified using polymerase chain reaction targeting the *17 kDa* antigen, *ompA*, and *gltA*, followed by sequencing for species identification and phylogenetic analysis. In total, 302 ticks belonging to four species (*Haemaphysalis flava*, *H. longicornis*, *Ixodes nipponensis*, and *Amblyomma testudinarium*) were collected between April and November 2022. The overall SFGR infection rate was 26.8% (81/302 patients). Both adult and nymphal ticks and the SFGR infection rate increased during April–May, reaching their peaks in June, followed by a marked decline in August and July, respectively. Phylogenetic analysis revealed three species (*R. monacensis, R. heilongjiangensis,* and *Candidatus* R. jingxinensis) of SFGR. Thus, our results emphasize the importance of tick surveys for the prevention and management of tick-borne rickettsiosis.

## 1. Introduction

Ticks are major blood-feeding arthropod vectors prevalent worldwide with three active feeding stages throughout their life cycle (larvae, nymphs, and adults). Tick-borne pathogens (TBPs) are acquired by the vector while feeding on an infected host [1,2]. These pathogens persist throughout transstadial transmission. Some pathogens, particularly *Rickettsia*, are transmitted transovarially from female ticks to their offspring [3]. Additionally, agricultural strategies, wildlife management, deforestation, and global warming contribute to the alteration of ecosystems, affecting the interaction between ticks and hosts as well as the circulation of TBPs. The resulting increased incidence of tick-borne diseases (TBDs) is now a global public health burden [4].

Rickettsiae, within the family Rickettsiaceae in the order Rickettsiales, are obligate intracellular bacteria that are transmitted to vertebrate hosts by arthropod vectors such as ticks, fleas, lice, and mites. *Rickettsia* species transmitted by ticks are mostly classified within the spotted fever group rickettsiae (SFGR), which causes tick-borne rickettsiosis in humans [5]. Tick species, including *Rhipicephalus*, *Ixodes*, *Amblyomma*, *Hyalomma*, *Haemaphysalis*, and *Dermacentor*, are recognized as SFGR vectors [6].

Human SFGR infections are primarily seen in America, the Mediterranean region, and East Asia [6]. According to the Centers for Disease Control and Prevention (CDC), the number of infected patients in the USA was between 3300 and 6200 per year between 2012 and 2019 [7]. In southeastern Brazil, 978 laboratory-confirmed SFGR cases were recorded from 2001 to 2018, of which 489 (50%) had a fatal outcome [8]. In addition, 5989 SFGR cases were identified in Italy between 2001 and 2015, with an average annual incidence of 0.88 per 100,000 [9]. Japan had 1765 cases of tick-borne rickettsiosis (Japanese spotted fever, *Rickettsia japonica*) between 2007 and 2016, 0.9% of which were fatal [10]. Moreover, a polymerase chain reaction (PCR) survey of patients with a recent tick bite between 2013 and 2016 at the Mudanjiang Forestry Central Hospital in China confirmed the presence of SFGR infections in 4.1% (111/2680) [11].

In the Republic of Korea (ROK), SFGR infection was first reported based on serological analyses of febrile patients between 1992 and 1993 [12]. The sera of patients with febrile illness between 1993 and 1999 were tested by molecular means, and sequence analysis revealed *Rickettsia conorii*, *R. japonica*, and *R. felis* [13]. The presence of *R. japonica* and *R. rickettsii* in *Haemaphysalis longicornis* was confirmed via PCR in 2003 [14]. Additionally, *R. monacensis* was isolated from *Ixodes nipponensis* in 2006 [15], and *R. japonica*, *R. monacensis*, and *Rickettsia* spp. were detected in *H. longicornis*, *H. flava*, and *I. nipponensis* collected from the southwestern provinces in 2013 [16]. Moreover, *R. monacensis* and *Rickettsia* spp. were isolated from patients in 2017 [2,17], and *R. monacensis* was also detected in the blood of patients in 2019 [18]. However, infected patients are no longer reported.

With the increasing global incidence of TBPs and tick-borne rickettsiosis, it is necessary to evaluate tick infections with SFGR. In the ROK, several efforts have been made to investigate the prevalence of SFGR in ticks; however, these efforts have been limited to certain areas. Furthermore, the pooling method for ticks did not fully reflect the exact infection rate, which is another limitation of previous studies in the ROK [19,20,21]. Therefore, we aimed to conduct the first nationwide molecular epidemiological survey of SFGR in ticks across the ROK to comprehensively understand the distribution of SFGR.

## 2. Materials and Methods

### 2.1. Tick Collection and Identification

**Figure 1:**
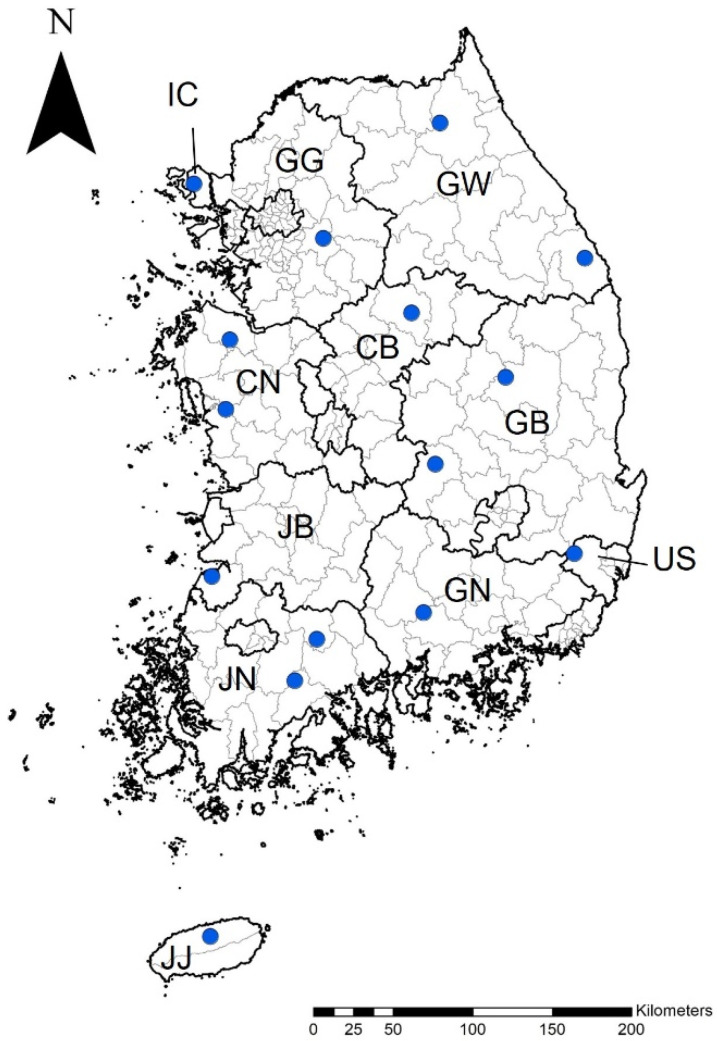
Geographic distribution of tick collection sites in this study. Collection sites in each metropolitan area or province are denoted by blue circles. This map was created using ArcGIS 9.0 software (Environmental Research System Institute, Redlands, CA, USA). Abbreviations: IC, Incheon (Ganghwa-gun); US, Ulsan (Ulju-gun) metropolitan area; GG, Gyeonggi (Gwangju-si); GW, Gangwon (Inje-gun, Samcheok-si); CB, Chungcheong-buk (Chungju-si); CN, Chungcheong-nam (Dangjin-si, Boryeong-si); GB, Gyeongsang-buk (Andong-si, Gimcheon-si); GN, Gyeongsang-nam (Jinju-si); JB, Jeolla-buk (Gochang-gun); JN, Jeolla-nam (Gokseong-gun, Boseong-gun); JJ, Jeju (Jeju-si) province.

Ticks were collected from 15 locations between April and November 2022: (1) in the metropolitan areas of Incheon (Ganghwa-gun) and Ulsan (Ulju-gun) and (2) in the provinces of Gyeonggi (Gwangju-si), Gangwon (Inje-gun, Samcheok-si), Chungcheong-buk (Chungju-si), Chungcheong-nam (Dangjin-si, Boryeong-si), Gyeongsang-buk (Andong-si, Gimcheon-si), Gyeongsang-nam (Jinju-si), Jeolla-buk (Gochang-gun), Jeolla-nam (Gokseong-gun, Boseong-gun), Jeju (Jeju-si) across the ROK (Figure 1). The ticks were collected using dry ice bait-collecting traps (Shin-Young Commerce System, Gyeonggi, Republic of Korea) and preserved in 70% ethanol. Subsequently, tick identification was performed via morphological analysis at the species level and developmental stage using a pictorial key [22]. 

From all of the tick samples collected from 15 locations, 302 ticks were systematically selected based on sampled region, month of collection, tick species, and developmental stages for individual molecular identification of SFGR. A maximum of 30 specimens per location was used, and to assess SFGR specificity among tick species, *Haemaphysalis longicornis*, the dominant species in the ROK [21,23], was limited to approximately 15 specimens per location. Efforts were made to diversify tick species, stages, and monthly abundance while adhering to these restrictions.

### 2.2. DNA Extraction

Each tick was individually placed in 2.8 mm bead tubes containing 400 µL of phosphate-buffered saline. The ticks were homogenized twice for 30 s at a speed of 4 m/s using a Precelly Evolution homogenizer (Bertin Technologies, Bretonneux, France) and centrifuged for 10 min at 12,000× *g*. The supernatant was used for DNA extraction along with a MagMAX™ DNA Multi-Sample Ultra 2.0 Kit (Applied Biosystems, Waltham, MA, USA) and the KingFisher Flex system (Thermo Fisher Scientific, Waltham, MA, USA) according to the manufacturer’s guidelines. The extracted DNA was stored at −20 °C until use. 

### 2.3. PCR Amplification

The ticks were screened first using the 17 kDa antigen coding gene (17 kDa), and positive samples were analyzed for outer membrane protein A gene (*ompA*) and citrate synthase gene (*gltA*) to detect SFGR. Only samples that yielded positive results for the three target genes were used for further analyses. The oligonucleotide primer sequences are listed in Table 1. Genomic DNA of *Rickettsia conorii* (Vircell, Granada, Spain) was used as a positive control for PCR. As a negative control, sterile water was included in each amplification trial.

PCR was performed using 20 µL of the AccuPower PCR PreMix (Bioneer Corp., Daejeon, Republic of Korea). Each PCR reaction consisted of 1 µL of each oligonucleotide primer (10 pmol/µL), 5 µL of genomic DNA as a template, and 13 µL of distilled water. A second PCR was performed using 1 µL of the primary PCR product as a template. Amplifications were carried out in the ProFlex PCR System (Thermo Fisher Scientific, Waltham, MA, USA), and the amplified products were subjected to electrophoresis in the automated QIAxcel^®^ system (QIAgen, Hilden, Germany). DNA extraction, PCR amplification, and automated electrophoresis were performed separately to prevent cross-contamination.

### 2.4. Phylogenetic Analysis

The PCR products were purified according to the protocol provides in the QIAquick PCR purification kit (QIAGEN, Ontario, Canada). The purified PCR products were sent to a commercial sequencing service (Bionics, Seoul, Republic of Korea) for sanger sequencing using the 3730XL DNA Analyzer (Applied Biosystems, Foster City, CA, USA). Sequences and reference sequences obtained from GenBank using nucleotide BLAST 2.15.0 (National Center for Biotechnology Information, NCBI) were aligned using CLUSTAL Omega (v.1.2.1). The nucleotide sequences of three genes were concatenated and aligned using MEGA v.11.0. Phylogenetic analyses were performed using the concatenated sequences in MEGA v.11.0. The phylogenetic tree was constructed using the neighbor-joining method based on the Kimura 2-parameter mode. The number on the branches indicates bootstrap percentages based on 1000 replications.

### 2.5. Statistical Analyses

A chi-square test was performed to analyze the association between the SFGR infection rate, tick species, and the region and season of tick collection. The analysis was performed using the GraphPad QuickCalcs website: https://www.graphpad.com/quickcalcs/chisquared1/, accessed on 30 January 2024. *p* < 0.05 was considered statistically significant.

## 3. Results

### 3.1. The Identification of Tick Species

Overall, 302 ticks were collected from 15 locations and classified into four species from three genera: *Haemaphysalis flava* (*n* = 83), *Haemaphysalis longicornis* (*n* = 204), *Ixodes nipponensis* (*n* = 4), *Amblyomma testudinarium* (*n* = 11). The dominant species were *H. longicornis* (67.5%), *H. flava* (27.5%), and *A. testudinarium* (3.6%) (Table 2). The ticks comprised 109 adult females (36.1%), 80 adult males (26.5%), and 113 nymphs (37.4%). The number of ticks collected was highest in June (39.1%, 118/302) followed by July (20.9%, 63/302).

### 3.2. Molecular Detection of SFGR in Ticks

Overall, 81 individual ticks tested positive for SFGR (infection rate, 26.8%). Three tick species were identified as having SFGR, with *H. longicornis* being the most common (77/204, 37.7%), while *H. flava* (3/83, 3.6%) and *I. nipponensis* (1/4, 25.0%) were only detected in three and one tick, respectively (Table 2). SFGR infection in *A. testudinarium* ticks was not detected in this study. SFGR infection rates in *H. longicornis* and *I. nipponensis* were significantly higher than those in *H. flava* (*p* < 0.0001 and *p* = 0.0461, respectively). During the developmental stages, SFGR was detected more often in adults (30.2%, 57/189) than in nymphs (21.2%, 24/113). Additionally, males (47.5%, 38/80) were confirmed to have a higher infection rate than females (17.4%, 19/109). 

Geographically, SFGR infection rates were the highest in the Incheon metropolitan area (Ganghwa-gun, 47.4%, 9/19), followed by Gangwon (Samcheok-si, 45.0%, 9/20), Chungcheong-buk (Chungju-si, 42.9%, 6/14), and Gyeongsang-buk provinces (Gimcheon-si, 40.0%, 8/20). The SFGR infection rate in the Incheon metropolitan area (Ganghwa-gun) was significantly higher than that in Gyeonggi (Gwangju-si) (*p* = 0.0004) and the Jeolla-nam (Gokseong-gun and Boseong-gun) provinces (*p* = 0.0046 and 0.0001), respectively (Table 3). 

**Figure 2:**
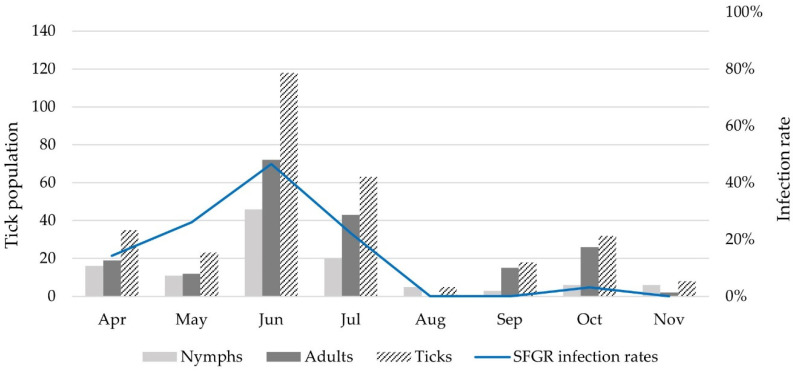
Seasonal distribution of tick populations and spotted fever group rickettsiae (SFGR) infection rates in ticks collected from the Republic of Korea.

According to month, the SFGR infection rates were 14.3% in April, 26.1% in May, 46.6% in June, 22.2% in July, and 3.1% in October. In particular, the SFGR infection rate was significantly higher in June than that in April and July–November (*p* < 0.0001 and *p* = 0.0002–0.0401), respectively (Figure 2 and Table S1).

### 3.3. SFGR Species Identification

**Figure 3:**
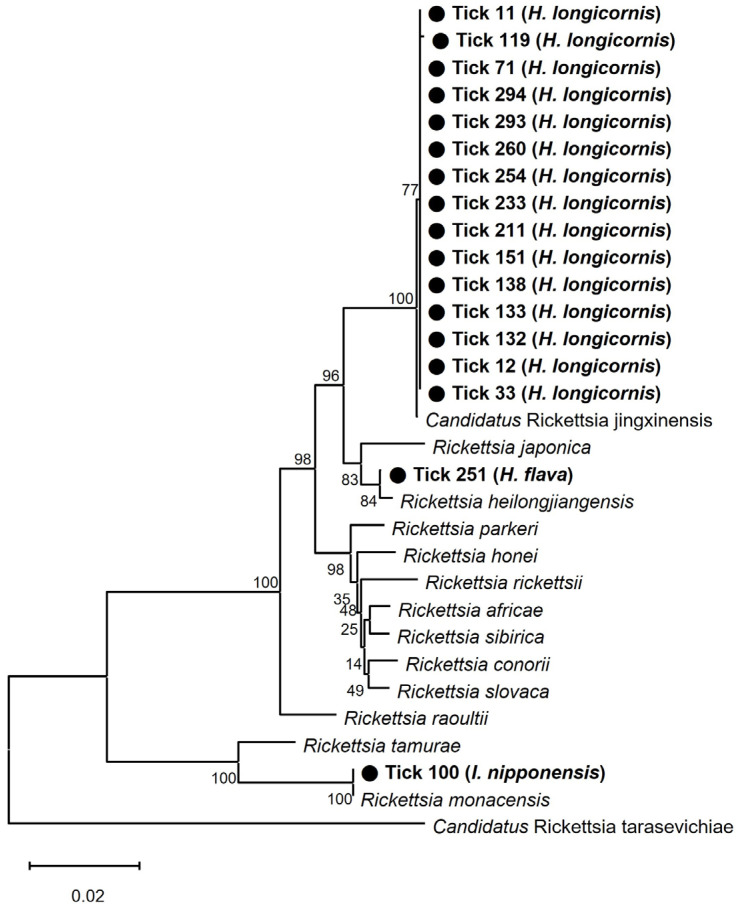
Phylogenetic analysis of spotted fever group rickettsiae (SFGR) using three (*17 kDa*, *ompA*, and *gltA*) concatenated sequences. The phylogenetic tree was constructed using the neighbor-joining method based on the Kimura two-parameter mode. The number on the branches indicates bootstrap percentages based on 1000 replications. A total of 1204 base pairs were included in the analysis.

The results of the phylogenetic analysis based on *17 kDa*, *ompA*, and *gltA* concatenated sequences are illustrated in Figure 3. The following representative sequences were selected for phylogenetic analysis without duplicate sequences: *17 kDa* (nine sequences, PP116492–500), *ompA* (11 sequences, PP116501–511), and *gltA* (three sequences, PP116512–514). They shared 100% identity with the *17 kDa* sequence previously reported for *R. monacensis* (LC379454.1), *R. heilongjiangensis* (CP112971.1), and *Candidatus* R. jingxinensis (MH932031.1) (Table S2). In addition, sequences obtained from *ompA* and *gltA* genes shared 99.6–99.8% and 99.2–100% identity with those previously reported for *R. monacensis* (MK613926.1 and MN630884.1), *R. heilongjiangensis* (MG906665.1 and MG906669.1), and *Candidatus* R. jingxinensis (MH932061.1 and MW114883.1) (Table S2).

One *H. flava* was infected with *R. heilongjiangensis* and two *H. flava* were infected with *Candidatus* R. jingxinensis. A total of 77 *H. longicornis* ticks were identified as *Candidatus* R. jingxinensis (Table 2). In addition, one *I. nipponensis* specimen was infected with *R. monacensis*.

## 4. Discussion

In the present study, we examined four tick species from three genera—*Haemaphysalis longicornis*, *H. flava*, *Ixodes nipponensis*, and *Amblyomma tetsudinarium*—and found that *H. longicornis* was the most abundant species (67.5%, 204/302) in the ROK. These results are consistent with those of previous studies in the ROK, which identified *H. longicornis* as the dominant tick species in various habitats [21,23].

We found SFGR infections in 26.8% (81/302) of the ticks collected. However, previous studies in the ROK on the SFGR infection rate in ticks showed a much lower minimum field infection rate (MFIR, assuming one positive tick/pool) of 0.9% (60/311 pools, 6484 ticks) in the southwestern region and 3.1% (88/284 pools, 2814 ticks) in the western region [18,19]. This discrepancy in infection rates could be attributed to the use of pooled ticks or individual ticks. In other countries, SFGR infection rates using individual ticks were 29.6% (931/3150) and 35.0% (75/214) in adults and nymphs in Germany and Japan, respectively [26,27]. Moreover, in northeastern China, the SFGR infection rate was 41.2% (103/250) in a survey of adults and nymphs [28].

In the present study, the infection rate in adult ticks (30.2%) was higher than that in nymphs (21.2%). This result is consistent with that of a previous study, which reported that adults had an SFGR infection rate 1.1–2.5 times higher than that of nymphs [26,29,30]. This difference is attributed to the transstadial transmission (horizontal) of SFGR throughout the developmental stages of ticks, resulting in its accumulation through transovarial transmission (vertical) [31,32,33].

Regarding the seasonal distribution of SFGR, the infection rates increased from April (14.3%) to May (26.1%) and peaked in June (46.6%). The peak decreased in July (22.2%), reached zero in August and September, and slightly increased in October (3.1%). A previous study in the ROK showed that SFGR in ticks was detected in 0.32% (13/341 pools, 4021 ticks), 0.42% (14/290 pools, 3303 ticks), 0.40% (17/355 pools, 4193 ticks), and 0.50% (1/108 pools, 200 ticks) MFIR in spring (March–May), summer (June–August), autumn (September–November), and winter (December–February), respectively, in Gwangju metropolitan area [20]. Unlike the findings of the present study, these results did not show significant differences in seasonal infection rates. It was assumed that each SFGR-positive tick pool contained only one positive tick, leading to a potential underestimation of the differences in infection rates compared with the actual rates.

However, the seasonal distribution of the results in this study appears to be similar to that in Central European countries with temperate climates characterized by four distinct seasons, similar to those of the ROK [34]. In a 3-year study conducted in Slovakia between 2011 and 2013, the distribution of SFGR in ticks between April and July (7.2–10.9%) was higher than that between August and October (2.6–8.1%) [29]. Furthermore, a study conducted in Germany between 2018 and 2019 revealed that the SFGR rate in August was significantly lower than that in all other months [26]. This seasonal pattern in the SFGR infection rate is presumed to be related to the life cycle of ticks in the ROK. In late summer and fall, the eggs hatch into larvae, which molt into nymphs in late fall. Unfed nymphs overwinter and emerge as active beings in the spring and early summer. In summer, adult females feed, lay eggs, and die [35]. Therefore, it is presumed that the SFGR infection rate begins to increase in spring and decreases in late summer, owing to the influence of nymph and adult populations. 

*Candidatus* Rickettsia jingxinensis was initially reported in *H. longicornis* in the northeastern region of China in 2016 and in subsequent studies performed in multiple provinces of China [36,37,38,39]. However, to date, it has only been identified in East Asia, including China and the ROK, and has mainly been detected in *H. longicornis* and *H. flava* ticks [36,38,40]. In this study, *Candidatus* R. jingxinensis was identified in 77 *H. longicornis* and 2 *H. flava*, accounting for the largest proportion (97.5%, 79/81 positive ticks) of SFGR detected. The *17 kDa*, *ompA*, and *gltA* sequences showed 99.6–100% identity with sequences previously reported in *H. longicornis* in China. Previously, *Candidatus* R. jingxinensis was reported in *H. longicornis* and *Amblyomma testudinarium* collected from humans in the ROK [24,41]. Although the pathogenicity of *Candidatus* R. jingxinensis remains unclear, it was observed in the ROK to be capable of infecting humans with the clinical characteristics of fever, erythematous rash, and eschar [2]. Thus, this species should be considered potentially pathogenic to humans. *Candidatus* R. jingxinensis is phylogenetically characterized as belonging to the subgroup around *R. japonica* [40]. In previous studies, *R. japonica* was detected in the sera of patients with febrile illnesses in the ROK between 1993 and 1999 [13]. Additionally, it was reported in *H. longicornis* collected from the Chungcheong Province (Chungju-si) in 2003 [16] and from the Chungcheong and Jeolla Provinces in the ROK in 2013 [19]. These findings suggest that a detailed phylogenetic analysis at the subgroup level, such as *Candidatus* R. jingxinensis, is necessary.

*Rickettsia monacensis*, an emerging human pathogen of SFGR, causes fever and erythematous rash with no eschar inoculation [42]. It is widely distributed in Europe, Asia, and Africa and is mainly transmitted by *Ixodes* spp. (especially *I. ricinus* and *I. nipponensis*) [5,18]. In the present study, *R. monacensis* was detected in one *I. nipponensis*. The *17 kDa* and *gltA* sequences showed high homology with the sequence detected in *I. nipponensis* in the ROK and Japan, and the *ompA* sequence was confirmed to have high homology (99.8%) with the sequence detected in a patient in the ROK in 2012 [2]. *R. monacensis* in the ROK was first reported in *I. nipponensis* collected from the Jeolla (Jeolla-buk and Jeolla-nam) provinces in 2013; since then, it has also been found in *I. nipponensis* ticks collected from the Gyeonggi, Gangwon, and Chungcheong (Chungcheong-buk and Chungcheong-nam) provinces [18,19,43,44].

In the present study, *Rickettsia heilongjiangensis* was detected in one *H. flava*. The *17 kDa*, *ompA*, and *gltA* sequences showed 99.2–100% identity with the sequences previously reported in humans and ticks in China. *R. heilongjiangensis* is a pathogenic agent of far-eastern tick-borne rickettsiosis. Reported cases occur in Russia, northern China, and Japan, and the main symptoms include rashes, eschars, and lymphadenopathy [42]. *Haemaphysalis* spp. (especially *H. concinna*, *H. flava*) and *Dermacentor silvarum* are its known primary vectors [5]. In the ROK, *R. heilongjiangensis* has been reported in *H. longicornis* and *H. flava* ticks collected from the Gyeonggi and Jeolla (Jeolla-buk and Jeolla-nam) provinces, respectively [19,45].

To our knowledge, this is the first nationwide study of SFGR in ticks in the ROK. In this study, questing ticks were collected using dry ice-baited traps. Previous studies suggest that variations in collection method performance are influenced by factors related to tick behavior, habitat characteristics, and climate [46,47]. Traditional surveillance methods for ticks typically involve the flagging method. However, this method is known to be vulnerable to sampling errors arising from vegetation type, sampler experience, and spatial distribution of ticks [23,48]. Therefore, we chose to collect ticks using dry ice-baited traps, which may result in fewer errors compared to the flagging method. Additionally, the methods is considered the most reliable method across various habitat types [23,47]. 

In the present study, a high SFGR infection rate was detected in adult and nymph ticks, which increased starting in April and reached a peak in June. However, the larvae were not included in this study. This is because the analysis of larvae has more limitations. First, as larval-stage ticks are the smallest in size, they contain comparatively fewer microbial nucleic acids. Furthermore, pooling methods may lead to a reduction in sensitivity (detection of true-positive samples) for pools with a large number of specimens and low infection prevalence [49]. These limitations may also contribute to the low infection rates of larval-stage ticks observed in previous studies [50]. Therefore, the present study focused on adults and nymphs to determine the infection rates of SFGR and tested individually. Additionally, this study selected some samples from the collected ticks and used them in analyses. Thus, owing to the limited number of populations by region in this study, further studies should involve a larger number of samples to increase the reliability of the geographical and seasonal distributions of the SFGR.

In this study, some samples were positive for the 17 kDa gene, but not the ompA or gltA gene, in the SFGR detection PCR. The four ticks (three *H. flava*, one *A. testudinarium*) confirmed to be positive for the 17 kDa gene were sequenced and identified as *R. raoultii* or *Candidatus* R. thierseensis (inconclusive from the 17 kDa gene). However, the ompA and gltA genes were not detected, and eventually we did not include these in the positive results. Therefore, surveillance of these species will also be needed.

Additionally, there is a possibility that the lack of awareness has led to misdiagnosis of other TBDs with non-specific symptoms, such as fever, headache, nausea, and vomiting. Consequently, the actual incidence of tick-borne rickettsiosis is predicted to be much higher [51,52]. Therefore, the data from this study strongly emphasize the importance of tick surveys for the prevention and management of tick-borne rickettsiosis.

## 5. Conclusions

To our knowledge, this is the first nationwide study of SFGR in ticks in the ROK. In the present study, a high SFGR infection rate was detected in adult (30.2%) and nymph (21.2%) ticks, which increased starting in April (14.3%) and reached a peak in June (46.6%). Phylogenetic analysis revealed three species (*Candidatus* R. jingxinensis, *R. monacensis*, and *R. heilongjiangensis*) of SFGR. Accounting for the largest proportion (97.5%, 79/81 positive ticks), *Candidatus* R. jingxinensis was identified in 77 *H. longicornis* and two *H. flava*. Additionally, *R. monacensis* was detected in one *I. nipponensis*, and *R. heilongjiangensis* was detected in one *H. flava*. These findings emphasize the importance of tick surveys for the prevention and management of tick-borne rickettsiosis.

## Figures and Tables

**Table 1:** Oligonucleotide primers used for detection of spotted fever group rickettsiae (SFGR).

Target Gene	Primer Name		Nucleotide Sequence (5’-3’)	Product Size (bp)	PCR Conditions	Reference
*17 kDa*	1st	Rr17k.1p	TTTACAAAATTCTAAAAACCAT	539	95 °C/5 m; 35 cycles: 95 °C/30 s, 57 °C/1 m, 72 °C/2 m; 72 °C/5 m	[24]
		Rr17k.539n	TCAATTCACAACTTGCCATT			
	2nd	Rr17k.90p	GCTCTTGCAACTTCTATGTT	450		
		Rr17k.539n	TCAATTCACAACTTGCCATT			
*ompA*	1st	R190.70F	ATGGCGAATATTTCTCCAAAA	634	94 °C/5 m; 40 cycles: 94 °C/30 s, 50 °C/30 s, 72 °C/1 m; 72 °C/5 m	
		RR190.701R	GTTCCGTTAATGGCAGCATCT			
	2nd	R190.70F	ATGGCGAATATTTCTCCAAAAA	535	94 °C/5 m; 5 cycles: 94 °C/30 s, 50 °C/30 s, 72 °C/30 s; 30 cycles: 94 °C/30 s, 54 °C/30 s, 72 °C/30 s; 72 °C/5 m	
		RR190.602R	AGTGCAGCATTCGCTCCCCCT			
*gltA*	1st	RpCS.780p	GACCATGAGCAGAATGCTTCT	479	95 °C/5 m; 35 cycles: 95 °C/30 s, 44 °C/30 s, 65 °C/2 m; 65 °C/5 m	[25]
		RpCS.1258n	ATTGCAAAAAGTACAGTGAACA			
	2nd	Rsfg.77p	GGGGGCCTGCTCACGGCGG	382	95 °C/5 m; 35 cycles: 95 °C/30 s, 48 °C/30 s, 65 °C/2 m; 65 °C/5 m	
		Rsfg.1258n	ATTGCAAAAAGTACAGTGAACA			

**Table 2:** Identified spotted fever group rickettsiae (SFGR) in ticks collected in the Republic of Korea.

Tick Species	Stages	Positive SFGR			Positive SFGR (%)	Total Positive SFGR (%)	*p*-Value
		*R. Monacensis*	*R. Heilong-Jiangensis*	*Candidatus* R. Jingxinensis			
*Haemaphysalis longicornis*	Female	0	0	19	19/73 (26.0)	77/204 (37.7)	**<0.0001**
	Male	0	0	36	36/54 (66.7)		
	Nymph	0	0	22	22/77 (28.6)		
*Haemaphysalis flava*	Female	0	0	0	0/30 (0.0)	3/83 (3.6)	-
	Male	0	1	0	1/24 (4.2)		
	Nymph	0	0	2	2/29 (6.9)		
*Amblyomma testudinarium*	Female	0	0	0	0/4 (0.0)	0/11 (0.0)	0.5216
	Male	0	0	0	0		
	Nymph	0	0	0	0/7 (0.0)		
*Ixodes* *nipponensis*	Female	0	0	0	0/2 (0.0)	1/4 (25.0)	**0.0461**
	Male	1	0	0	1/2 (50.0)		
	Nymph	0	0	0	0		
Total		1 (0.3)	1 (0.3)	79 (26.2)	81/302 (26.8)		-

A chi-square test was used to analyze the differences in SFGR infection rates among tick species; significant values are indicated in bold (*p* < 0.05).

**Table 3:** Geographical distribution of spotted fever group rickettsiae (SFGR)-positive ticks collected in the Republic of Korea.

	Region		Tick Species	No. of Ticks	Positive SFGR (%)	*p*-Value
Metropolitan area	Incheon	Ganghwa-gun	*H. longicornis*	15	9/19(47.4)	-
			*H.* *flava*	4		
	Ulsan	Ulju-gun	*H. longicornis*	15	8/26 (30.8)	0.2566
			*H.* *flava*	5		
			*A. testudinarium*	5		
			*I. nipponensis*	1		
Provinces	Gyeonggi	Gwangju-si	*H. longicornis*	10	0/20 (0.0)	**0.0004**
			*H.* *flava*	10		
	Gangwon	Inje-gun	*H. longicornis*	13	2/18 (11.1)	0.0159
			*H.* *flava*	5		
		Samcheok-si	*H. longicornis*	15	9/20 (45.0)	0.8821
			*H.* *flava*	5		
	Chungcheong-buk	Chungju-si	*H. longicornis*	10	6/14 (42.9)	0.7970
			*H.* *flava*	3 *		
	Chungcheong-nam	Dangjin-si	*H. longicornis*	15	7/24 (29.2)	0.2201
			*H.* *flava*	9		
		Boryeong-si	*H. longicornis*	13	5/17 (29.4)	0.2699
			*H.* *flava*	4		
	Gyeongsang-buk	Andong-si	*H. longicornis*	10	6/17 (35.3)	0.4632
			*H.* *flava*	7		
		Gimcheon-si	*H. longicornis*	15	8/20 (40.0)	0.6428
			*H.* *flava*	1		
			*A. testudinarium*	3		
			*I. nipponensis*	1		
	Gyeongsang-nam	Jinju-si	*H. longicornis*	14	6/17 (35.3)	0.4632
			*H.* *flava*	1		
			*I. nipponensis*	12 *		
	Jeolla-buk	Gochang-gun	*H. longicornis*	15	6/19 (31.6)	0.3194
			*H.* *flava*	1		
			*A. testudinarium*	3		
	Jeolla-nam	Gokseong-gun	*H. longicornis*	16	2/23 (8.7)	**0.0046**
			*H.* *flava*	7		
		Boseong-gun	*H. longicornis*	14	0/24 (0.0)	**0.0001**
			*H.* *flava*	10		
	Jeju	Jeju-si	*H. longicornis*	14	7/24 (29.2)	0.2201
			*H.* *flava*	10		
	Total			302	81 (26.8)	

A chi-square test was used to analyze the difference in SFGR infection rates among tick collection sites; significant values are indicated in bold (*p* < 0.05). * *Rickettsia heilongjiangensis* and *R. monacensis* were detected in one tick collected in Chungcheong-buk (Chungju-si) and Gyeongsang-nam (Jinju-si), respectively. In the other 79 ticks of positive SFGR, *Candidatus* R. jingxinensis was detected.

## Data Availability

Data supporting the conclusions of this article are included within the article. The newly generated sequences were submitted to the GenBank database under the accession numbers PP116492–PP116511. The datasets used and/or analyzed during the present study are available from the corresponding author upon reasonable request.

## Supplementary Materials

The following supporting information can be downloaded at: https://www.mdpi.com/article/10.3390/pathogens13070575/s1, Table S1: Distribution of detected spotted fever group rickettsiae (SFGR) in the monthly and developmental stages of ticks collected in the Republic of Korea; Table S2: GenBank accession numbers of spotted fever group rickettsiae (SFGR) gene sequences used in the phylogenetic analysis.
