# Supplementary material for: Molecular Identification of Spotted Fever Group Rickettsiae in Ticks in the Republic of Korea
Source: Pathogens. 2024 Jul 10;13(7):575. doi: 10.3390/pathogens13070575 (PMC11280320; doi:10.3390/pathogens13070575)
Supplement: Supplementary file 1 [file pathogens-13-00575-s001.zip › pathogens-3081953-supplementary.pdf]

Supplementary Materials

Table S1. Distribution of detected spotted fever group Rickettsiae (SFGR) in the monthly and developmental stages of ticks collected in the Republic of Korea.

Month	Tick stage	Ticks	Positive SFGR (%)	*P* - value
April	Female	14	2 (14.3)	**0.0006**
	Male	5	1 (20.0)	
	Nymph	16	2 (12.5)	
	**Total**	**35**	**5 (14.3)**	
May	Female	8	0 (0.0)	0.0692
	Male	4	2 (50.0)*	
	Nymph	11	4 (36.4)	
	**Total**	**23**	**6 (26.1)**	
June	Female	37	13 (35.1)	-
	Male	35	27 (77.1)	
	Nymph	46	15 (32.6)	
	**Total**	**118**	**55 (46.6)**	
July	Female	27	4 (14.8)	**0.0013**
	Male	16	7 (43.8)	
	Nymph	20	3 (14.8)	
	**Total**	**63**	**14 (22.2)**	
August	Female	-	-	**0.0401**
	Male	-	-	
	Nymph	5	0 (0.0)	
	**Total**	**5**	**0 (0.0)**	
September	Female	7	0 (0.0)	**0.0002**
	Male	8	0 (0.0)	
	Nymph	3	0 (0.0)	
	**Total**	**18**	**0 (0.0)**	
October	Female	14	0 (0.0)	**< 0.0001**
	Male	12	1 (8.3)*	
	Nymph	6	0 (0.0)	
	**Total**	**32**	**1 (3.1)**	
November	Female	2	0 (0.0)	**0.0101**
	Male	-	-	
	Nymph	6	0 (0.0)	
	**Total**	**8**	**0 (0.0)**	
	**Total**	**302**	**81 (26.8)**	

A chi-square test was used to analyze the difference in the SFGR infection rate among the tick collection months; significant values are shown in bold ($P < 0.05$).

* *Rickettsia heilongjiangensis* and *R. monacensis* were detected in one male tick collected in April and October, respectively. In the other 79 ticks of positive SFGR, *Candidatus* R. jingxinensis was detected.

Table S2. GenBank accession numbers of spotted fever group Rickettsiae (SFGR) gene sequences used in the phylogenetic analysis.

Species	*17 kDa*	*ompA*	*gltA*
R. africae	CP001612.1	U43790.1	U59733.1
R. conorii	AE006914.1	U43791.1	U59730.1
R. heilongjiangensis	CP112971.1	MG906665.1	MG906669.1
R. honei	AF027124.1	AF018075.1	AF018074.1
R. japonica	AP011533.1	D28766.1	U59724.1
R. monacensis	LC379454.1	MK613926.1	MN630884.1
R. parkeri	CP003341.1	U43802.1	MN027564.1
R. raoultii	MH212177.1	KR608786.1	DQ365804.1
R. rickettsii	MH212177.1	MF988095.1	KF742602.1
R. sibirica	AF445384.1	U43807.1	U59734.1
R. slovaca	CP002428.1	U43808.1	U59725.1
R. tamurae	AB812550.1	LC388793.1	AF394896.1
Candidatus R. jingxinensis	MH932031.1	MH932061.1	MW114883.1
Candidatus R. tarasevichiae	KX365195.1	LC379461.1	KT899085.1
